# Supplementary material for: The burden of traditional neonatal uvulectomy among admissions to neonatal intensive care units, North Central Ethiopia, 2019: A triangulated crossectional study
Source: PLoS One. 2020 Jul 9;15(7):e0234866. doi: 10.1371/journal.pone.0234866 (PMC7347129; doi:10.1371/journal.pone.0234866)
Supplement: S1 File — (DOCX) [file pone.0234866.s001.docx]

**English version of the questionnaire**

Questionnaire code………….

**General instruction**

1. For multiple choice questions, choose the best answer
2. If your answer is not listed among alternatives, please tell your own answer for the data collector.

**Part one**

I. Socio-demographic characteristics

| S/N | Factor | Response |
| --- | --- | --- |
| 100 | Residence | 1. Urban 2. Rural |
| 101 | Maternal age | **______ (years)** |
| 102 | Marital status | 1. Married 2. Divorced 3. Widowed |
| 103 | Parity | 1. Primiparous 2. Multiparous |
| 104 | Maternal educational status | 1. Unable to read and write 2. Primary education 3. Secondary education 4. College/university |
| 105 | Husband’s educational status | 1. Unable to read and write 2. Primary education 3. Secondary education 4. College/university |
| 106 | Maternal occupation | 1. Civil servant 2. Merchant 3. House wife |
| 107 | Average monthly income | **________________ ($ US)** |

**Part II**: **Obstetrics related factors**

| **S/N** | **Factor** | **Response** |
| --- | --- | --- |
| 200 | ANC follow up | 1. Yes 2. No |
| 201 | Number of ANC visits | _____________ |
| 202 | Accompanied by spouse to ANC | 1. Yes 2. No |
| 203 | Antenatal counseling of traditional neonatal uvulectomy | 1. Yes 2. No |
| 204 | Antenatal couple counseling of traditional neonatal uvulectomy | 1. Yes 2. No |
| 205 | If yes to Q_4_ and/Q_5,_ what were you counseled? | 1. Adverse effects of traditional neonatal uvulectomy 2. Immediate modern health care seeking during perception of elongated uvula 3. The presence of modern medicine for elongated uvula 4. Benefits of uvula 5. Other (_____) |
| 206 | Ever had bad obstetrics history | 1. Yes 2. No |
| 207 | If yes, which of the following? | 1. A. Neonatal death 2. B. Child death 3. C. Still birth 4. D. Abortion 5. E. IUFD 6. F. Other (________) |
| 208 | Place of delivery | 1. Health institution 2. Home |
| 209 | PNC visit | 1. Yes 2. No |
| 210 | Number of PNC visits | 1. 1^st^ PNC visit (Within 24 hours of birth) 2. 2^nd^ PNC visit (1 – 7) days after birth |
| 211 | Accompanied by spouse to PNC | 1. Yes 2. No |
| 212 | Postnatal counseling about traditional neonatal uvulectomy | 1. Yes 2. No |
| 213 | Postnatal couple counseling about traditional neonatal uvulectomy | 1. Yes 2. No |
| 214 | If yes to Q_13_ and/Q_14,_ what were you counseled? | 1. Adverse effects of traditional neonatal uvulectomy 2. Immediate modern health care seeking during perception of elongated uvula 3. The presence of modern medicine for elongated uvula 4. Benefits of uvula |

**Part III: Neonatal characteristics**

| **S/N** | **Factor** | **Response** |
| --- | --- | --- |
| 300 | Sex | 1. Male 2. Female |
| 301 | Gestational age at birth | _______(weeks) |
| 302 | Post natal age at admission | _______ (days) |
| 303 | Birth weight | _______ (grams) |
| 304 | Medical diagnosis @ admission | 1. Hypothermia 2. Early onset neonatal sepsis 3. Late onset neonatal sepsis 4. Prematurity 5. Perinatal asphyxia 6. Hypoglycemia 7. Congenital defect 8. Other ( ___ ) |

**Part IV: Maternal knowledge of neonatal uvula and traditional uvulectomy**

| **S/N** | **Factor** | | **Response** | |
| --- | --- | --- | --- | --- |
| 400 | Can you mention benefits of uvula? | 1. Yes 2. No | |  |
| 401 | If yes to Q_1_, what are the benefits of uvula? | 1. Preventing aspiration while swallowing breast milk 2. Lubricating oropharyngeal mucosa 3. Serving for language communication 4. Boosting immunological function 5. prevention of breast milk regurgitation through the neonatal nose 6. other ( _____ ) | |  |
| 402 | Can you mention the adverse effects of traditional neonatal uvulectomy? | 1. Yes 2. No | |  |
| 403 | If yes to Q_3_, what are these adverse effects? | 1. Transmission of communicable infections (HIV, Hep B) 2. Hemorrhage 3. Tetanus 4. Pharyngeal dryness 5. Aspiration 6. Pain 7. Change in voice 8. disturbance in sleep pattern 9. Regurgitation of breast milk from the nostril 10. Others ( ______ ) | |  |
| 404 | Do you have prior history of traditional neonatal uvulectomy? | 1. Yes 2. No | |  |

**Part V: Burden and attributes of traditional uvulectomy at admission**

| S/N | Factor | Response |
| --- | --- | --- |
| 500 | Was your index neonate done  uvulectomy? (If your answer is  No, please stop here.) | 1. Yes 2. No |
| 501 | When was uvulectomy done? | ___ (days after birth) |
| 502 | Who did influence you to practice  traditional neonatal uvulectomy to  your neonate? | 1. Traditional uvulectomists 2. Family 3. Traditional birth attendants 4. Friends 5. Maternal own decision 6. Other (specify …) |
| 503 | What was the primary  postuvulectomy complication at  admission? | 1. Sepsis 2. Anemia 3. Neck swelling 4. Tongue and oropharyngeal injury 5. Others (___) |

## Amharic version of the questionnaire

**አጠቃላይመመሪያ**

**1. ለምርጫ ጥያቄዎች ከቀረቡት አማራጮች አንዱን ይምረጡ፡፡**

**2. ከቀረቡት አማራጮች መካከል መልስ ካላገኙ የራስዎን መልስ ለመረጃ ሰብሳቢዉ ይንገሩት፡፡**

**ክፍል አንድ፡ አጠቃላይ ሁኔታን የሚመለከቱ ጥያቄዎች**

| **ተ.ቁ** | **የቃለ መጠይቁ ጥያቄዎች** | **መልስ** |
| --- | --- | --- |
| **100** | የመጡበት አካባቢ | 1. ከተማ 2. ገጠር |
| **101** | የእናት እድሜ | **-----------------** (አመት) |
| **102** | የጋብቻ ሁናቴ | 1. ያገባች 2. ያላገባች 3. አግብታ የፈታች 4. ባሏ የሞተባት |
| **103** | ስንት ልጆች ወልደዋል? | 1. አንድ ብቻ 2. ሁለትና ከዛ በላይ |
| **104** | የእናትዬዋ የትምህርት ደረጃ | 1. ማንበብና መፃፍ አትችልም 2. የመጀመሪያ ደረጃ ትምህርት 3. የሁለተኛ ደረጃ ትምህርት 4. ዲፕሎማና ከዛበላይ |
| **105** | የአባትየዉ የትምህርት ደረጃ | 1. ማንበብና መፃፍ አይችልም 2. የመጀመሪያ ደረጃ ትምህርት 3. የሁለተኛ ደረጃ ትምህርት 4. ዲፕሎማና ከዛበላይ |
| **106** | የእናትዬዋ ስራ | 1. የቤት እመቤት 2. የመንግስት ሰራተኛ 3. የግል ስራ የምትሰራ 4. ተማሪ 5. አርሶአደር |
| **107** | አማካኝ ወርሃዊ ገቢ | ---------- **($US)** |

**ክፍል ሁለት፡ የሙቀት መጠን እና ለሙቀት መቀነስ የሚያጋልጡ ተዛማጅ ምክንያቶችን መጠየቂያ**

| ተ.ቁ | የቃለ መጠይቁ ጥያቄዎች | መልስ |
| --- | --- | --- |
| 200 | የቅድመ-ወሊድ ክትትል አድርገዋል? | 1. አዎ 2. አይደለም |
| 201 | መልስዎ አዎ ከሆነ፣ ስንት ጊዜ? | ----------- |
| 202 | በቅድመ-ወሊድ ክትትል ጊዜ ከባለቤትዎ ጋር ነበሩን? | 1. አዎ 2. አይደለም |
| 203 | በቅድመ -ወሊድ ክትትል ጊዜ ስለ ባህላዊ እንጥል ማስቆረጥ የምክር አገልግሎት አግኝተዋልን? | 1. አዎ 2. አይደለም |
| 204 | መልስዎ አዎ ከሆነ የምክር አገልግሎቱን ያገኙት ከባለቤትዎ ጋር ነዉን? | 1. አዎ 2. አይደለም |
| 205 | መልስዎ አዎ ከሆነ ያገኙት የምክር አገልግሎት ስለምን ነዉ ? | 1. ባህላዊ እንጥል ማስቆረጥ በጨቅላ ህፃናት ጤና ላይ ስለሚያስከትለዉ ጉዳት 2. እንጥል በዘመናዊ መንገድ ማዳን እንደሚቻል 3. ስለእንጥል ጥቅም 4. ሌላ ካለ ይጠቀስ |
| 206 | በቀደምት እርግዝናዎ ዉጤት ላይ ችግር ነበርን? | 1. አዎ 2. አይደለም |
| 207 | መልስዎ አዎ ከሆነ የትእኛዉ ነዉ? | 1. የጨቅላ ህፃን ሞት 2. የህፃን ሞት 3. በወሊድ ጊዜ የሞተ 4. ውርጃ 5. ማህፀን ዉስጥ ሞቶ የተወለደ 6. ሌላ ካለ ይጠቀስ |
| 208 | የወለዱበት ቦታ | 1. ቤት ዉስጥ 2. በጤና ተቋም |
| 209 | **የ**ድህረ-ወሊድ ክትትል አድርገዋልን; | 1. አዎ 2. አይደለም |
| 210 | መልስዎ አዎ ከሆነ ስንት ጊዜ? | ­­­­­­­­­­­­­-------------- |
| 211 | በድህረ-ወሊድ ክትትል ጊዜ ከባለቤትዎ ጋር ነበሩን? | 1. አዎ 2. አይደለም |
| 212 | በድህረ-ወሊድ ክትትል ጊዜ ስለ ባህላዊ እንጥል ማስቆረጥ የምክር አገልግሎት አግኝተዋልን? | 1. አዎ 2. አይደለም |
| 213 | መልስዎ አዎ ከሆነ የምክር አገልግሎቱን ያገኙት ከባለቤትዎ ጋር ነዉን? | 1. አዎ 2. አይደለም |
| 214 | መልስዎ አዎ ከሆነ ያገኙት የምክር አገልግሎት ስለምን ነዉ ? | 1. ባህላዊ እንጥል ማስቆረጥ በጨቅላ ህፃናት ጤና ላይ ስለሚያስከትለዉ ጉዳት 2. እንጥልን በዘመናዊ መንገድ ማዳን እንደሚቻል 3. ስለእንጥል ጥቅም 4. ሌላ ካለ ይጠቀስ (_____) |

**ክፍል ሶስት፡የጨቅላ ህፃናት መለያዎች**

| ተ.ቁ | የቃለ መጠይቁ ጥያቄዎች | መልስ |
| --- | --- | --- |
| 300 | የጨቅላ ህፃኑ ጶታ | 1. ወንድ 2. ሴት |
| 301 | ጨቅላ ህፃኑ የተወለደበት የእርግዝና ጊዜ ስንት ነዉ? | ________(በሳምንት) |
| 302 | የጨቅላ ህፃኑ እድሜ ስንት ነዉ? | __________(በቀን) |
| 303 | ጨቅላ ህፃኑ ሲዎለድ የነበረዉ ክብደት ስንት ነዉ? | __________(በግራም) |

**ክፍል አራት: እናቶች ስለ ጨቅላ ህፃናት እንጥል እና እንጥልን በባህላዊ መንገድ ማስቆረጥ ያላቸዉን እዉቀት የሚመለከት**

| ተ.ቁ | የቃለ መጠይቁ ጥያቄዎች | መልስ |
| --- | --- | --- |
| 400 | የእንጥልን ጥቅም መዘርዘር ይችላሉ? | 1. አዎ 2. አይደለም |
| 401 | መልስዎ አዎ ከሆነ፣ ይዘርዝፘቸዉ:: | 1. ትንታን መከላከል 2. አፍ እና ላንቃን እንዳይደረቅ መከላከል 3. ለድምፅ ጥራት 4. ከበሺታ የመከላከል አቅምን ለመጨመር 5. የጡት ወተት ወደ አፍንጫ እንዳይመለስ 6. ሌላ ካለ ይጠቀስ ( _____ ) |
| 402 | እንጥልን በባህላዊ መንገድ ማስቆረጥ በጨቅላ ህፃናት ጤና ላይ ስለሚያስከትለዉ ጉዳት መዘርዘር ይችላሉ? | 1. አዎ 2. አይደለም |
| 403 | መልስዎ አዎ ከሆነ፣ ይዘርዝፘቸዉ:: | 1. ለተላላፊ በሺታዎች ያጋልጣል (HIV, Hep B) 2. ለደም መፍሰስ ይዳርጋል 3. ለመንጋጋ በሺታ ያጋልጣል 4. የአፍ እና ላንቃ መድረቅ 5. ትንታ 6. የህመም ስሜት 7. የድምፅ ጥራት መቀየር 8. የእንቅልፍ ኡደት መዛባት 9. የጡት ወተት ወደ አፍንጫ መመለስ 10. ሌላ ካለ ይጠቀስ ( _____ ) |
| 404 | ከዚህ በፊት በባህላዊ መንገድ እንጥል አስቆርጠዉ ያዉቃሉ? | 1. አዎ 2. አይደለም |

**ክፍል አምስት: ከባህላዊ እንጥል ማስቆረጥ ጋር በተያያዘ ችግር ምክንያት ለህክምና ወደ ጨቅላ ህፃናት ፅኑ ህሙማን ክፍል የገቡ ህፃናትን የሚመለከቱ ጥያቄዎች**

| ተ.ቁ | የቃለ መጠይቁ ጥያቄዎች | | መልስ |
| --- | --- | --- | --- |
| 500 | የጨቅላ ህፃንዎን እንጥል አስቆርጠዋልን?(መልስዎ አይደለም ከሆነ፣, ከዚህ ጋ ያቁሙ) | | 1. አዎ 2. አይደለም |
| 501 | እንጥል ያስቆረጡ መቼ ነዉ? | | ___ (ከተወለደ በኋላ በቀናት) |
| 502 | የጨቅላ ህፃንዎን እንጥል ለማስቆረጥ የገፋፋዎት ማን ነዉ? | | 1. ባህላዊ እንጥል ቆራጮች 2. ቤተሰብ 3. የልምድ አዋላጆች 4. ጓደኞች 5. የራስ ዉሳኔ 6. ሌላ ካለ ይጠቀስ ( _____ ) |
| **Checklist about neonatal health problems at admission** | | | |
| 600 | Medical diagnosis @ admission | 1. Hypothermia 2. Early onset neonatal sepsis 3. Late onset neonatal sepsis 4. Prematurity 5. Perinatal asphyxia 6. Hypoglycemia 7. Congenital defect 8. Other ( ___ ) | |
| 601 | What was the primary postuvulectomy complication at admission (for neonates who were done uvulectomies)? | 1. Sepsis 2. Anemia 3. Neck swelling 4. Tongue and oropharyngeal injury 5. Others (___) | |
