# Supplementary material for: The burden of traditional neonatal uvulectomy among admissions to neonatal intensive care units, North Central Ethiopia, 2019: A triangulated crossectional study
Source: PLoS One. 2020 Jul 9;15(7):e0234866. doi: 10.1371/journal.pone.0234866 (PMC7347129; doi:10.1371/journal.pone.0234866)
Supplement: S2 File — (DOCX) [file pone.0234866.s002.docx]

**Interview guide**

**English version of the interview guide**

1. What are the enablers of traditional neonatal uvulectomy?
2. What is your opinion about the adverse effects of traditional neonatal uvulectomy?
3. How could it be possible to prevent traditional neonatal uvulectomy?

**Amharic version of the interview guide**

1. **የጨቅላ ህፃናትን እንጥል በባህላዊ መንገድ ለማስቆረጥ የሚገፋፉ ሁናቴዎች ምንድን ናቸዉ?**
2. **ባህላዊ እንጥል ማስቆረጥ በጨቅላ ህፃናት ጤና ላይ ስለሚያስከትለዉ ጉዳት ያለዎት አስተያየት ምንድን ነዉ?**
3. **ባህላዊ እንጥል ማስቆረጥን በምን መንገድ መከላከል ይቻላል ብለዉ ያስባሉ?**
