# Supplementary material for: The burden of traditional neonatal uvulectomy among admissions to neonatal intensive care units, North Central Ethiopia, 2019: A triangulated crossectional study
Source: PLoS One. 2020 Jul 9;15(7):e0234866. doi: 10.1371/journal.pone.0234866 (PMC7347129; doi:10.1371/journal.pone.0234866)
Supplement: Attachment — Submitted filename: Review PO.docx [file pone.0234866.s003.docx]

Thank you for giving me the opportunity to review this paper discussing traditional practice of neonatal uvulectomy which, according to the authors, is often unsupervised and unscientific and a potentially dangerous cultural malpractice. The authors have addressed this issue which seems very important to address as proper education could potentially minimize the harmful consequences of these uvulectomies.

Throughout the article traditional uvulectomy is referred to as “… the malpractice” which is not objective, so would suggest to change to ‘traditional uvulectomy’, unless it flows naturally from the sentence before like the last line of the introduction (“Nonetheless, there is no current regional and even national data about the proportion, associated factors and reasons of this malpractice.”).

Reference 10 and 25 should be mentioned in the text, not as reference. The references in general should be thoroughly revised as they do not meet the APA standards.

My most important comment/concern is the presentation of the study results. For me it is unclear how many uvulectomies were done, if there were potentially uvulectomies missed (bias) and how the admission percentages were calculated. From the abstract and methods it seems that 422 woman were admitted to hospital, but then in the discussion there is a 15% admission rate mentioned? A flow diagram with inclusions, exclusions and complication rates would be helpful. *“Male sex, home delivery and prior history of traditional uvulectomy were significantly associated with increased odds of traditional neonatal uvulectomy.”* Is this associated with the uvulectomy itself or with the **complications of uvulectomy**?

*“Mixed type (quantitative supplemented with qualitative) hospital based cross sectional study was conducted. Phenomenological study design was employed for the qualitative part. All postnatal mothers whose age ≥ 18 years and lived at least six months in the study area prior to the study and visiting Neonatal Intensive Care Unit (NICU) during the study were eligible. However, abandoned neonates (those neonates left in NICU without mothers), mothers with critical illness or any difficulty of talking/listening, those having psychiatric disorders and known medical problems were excluded since they were not mentally and physically capable of being interviewed. Non volunteer mothers were also excluded.”* This doesn’t sound free of bias, it seems that there might be a lot of cases missed and that only the potential ‘tip of the iceberg’ has been investigated.

The sample size calculations are based on quite a few assumptions and the sample size of the qualitative study seems very small (n=8).

*“Regarding counseling of traditional neonatal uvulectomy, 103 (63.0%) mothers were given antenatal counseling. Besides, 44 (42.7%) of the mothers were given the counseling together with their husbands.”* This should be addressed in the discussion. What is the assumption here? That if husbands are present they are more or less likely to comply with counselling?

*“Neonates born to parents who were couple counseled of traditional neonatal uvulectomy during antenatal period were 94.7% less likely to be victim as compared to those neonates born to parents who weren’t couple counseled [AOR= 0.053; 95% CI: 0.01, 0.35].”* This is a very important conclusion of the paper that should be stressed more (i.e. included in abstract and conclusion). On which data is this conclusion based?

*‘After I delivered at home, all the men and women who helped me during the birth were dealing with the essence of contacting traditional uvulectomy practitioners if my kid becomes irritable despite good breastfeeding. This is because nowadays elongated uvula is chiefly characterized by irritability rather than decreased breastfeeding. Then, I experienced traditional uvulectomy when I was in trouble of the kid’s spontaneous crying despite its successful breastfeeding’* – what does this mean? The statements of the qualitative responses are hard to interpret in general. Could there be a better way of including this information? Most of the statements are very valuable, but they are rather long. I would suggest a major revision of the paper to make this information more easily digestible.

Table 5 says *“Neonatal age at uvulectomy n=67”*; does this mean that only 67 of the 422 mothers answered this question?

Forty percent of information about traditional uvulectomy comes from traditional uvulectomy surgeons (!). Is there a way to counsel them and if so, could this be discussed in the discussion?

*The likelihood of traditional uvulectomy among neonates whose mothers and fathers received counseling of traditional uvulectomy during postnatal visit was 89.9% lower than those whose parents weren’t couple counseled [AOR= 0.101; 95% CI: 0.02, 0.65]. This finding was supported by a key informant who said: ‘Just in front of our kid at post natal room, my husband and me were advised of the life threatening septic and hemorrhagic complications of traditional neonatal uvulectomy which we had never known before. It was heart touching to hear the advice in front of our kid. Since then, we promised never to experience traditional uvulectomy for our kid’.* This seems to contradict with the information given in Table 2. In Table 2, 63% received neonatal counselling, yet they all had traditional uvulectomy.

With all comments taken into account, the discussion should be revised and resubmitted for further review. Most importantly, the results and consequences of current practice should be discussed. There are many potential limitations including several biases (selection bias, inclusion bias, reporting bias, …) that should be discussed.

Other minor comments:

Introduction:

- “There are divergent views to the reason as well as its overall benefit in these countries.” This statement seems taken out of the air without references or examples.

Table 1:

- I assume the 4^th^ line under husband’s educational status should say college/university instead of secondary education twice.

Results:

- *“Neonates’ failure to breast feed 16 (23.9%) was the most reported indicator of elongated uvula (****F****igure 2).”* What do the authors mean by this?

Table 6:

- I would suggest to remove the significance from the bottom and just add an extra column with p-values.

Discussion:

- … tried to address … suggest to change to ‘addressed the’

Figures:

- The Figures are not very informative to me. Please reconsider the information provided.

Textual suggestions:

- In general, the quality of the English is poor, I would suggest a language revision by a preferably native English speaker.
- ~~(disadvantages)~~ suggest to remove
- Top of page 5 ‘literatures” suggest to change to ‘studies’
- ? DTGH – change to ‘the hospital’
- Punctuation should be consistently checked; consistency of capitals (i.e. Table 1, Figure 1, etc.) and (n=xx, xx%) in correct form.
