# Supplementary material for: The burden of traditional neonatal uvulectomy among admissions to neonatal intensive care units, North Central Ethiopia, 2019: A triangulated crossectional study
Source: PLoS One. 2020 Jul 9;15(7):e0234866. doi: 10.1371/journal.pone.0234866 (PMC7347129; doi:10.1371/journal.pone.0234866)
Supplement: Attachment — Submitted filename: Response to Reviewers.docx [file pone.0234866.s005.docx]

**Response letter**

**Dear academic Editor (Ju Lee Oei)**

*After going through the entire revised version manuscript, you forwarded your constructive editorial comments which we missed to touch. Therefore, we are glad enough to express our sincerest thanks for your constructive comments that could help improve novelty of our effort.*

Editorial comment: The manuscript is much improved. However, please edit it for minor grammatical details and presentation. For example, two decimal points are not needed in many of the results.

Authors’ response: It is really a comment of relevance in increasing readability of the paper through conveying the key messages in an intelligible and easily understandable language. Therefore, the manuscript is again revised for the required grammatical details and presentation of results. Results are now consistently presented with two decimal points which could show higher precision than a single decimal point.

All the improved changes are incorporated to the second revised version manuscript as shown by the yellow highlighted text.

Thank you in advance!

Wubet Alebachew
